# Hydrology controls dissolved organic matter export and composition in an Alpine stream and its hyporheic zone

**DOI:** 10.1002/lno.10232

**Published:** 2015-12-01

**Authors:** Christina Fasching, Amber J. Ulseth, Jakob Schelker, Gertraud Steniczka, Tom J. Battin

**Affiliations:** ^1^ Department of Limnology and OceanographyUniversity of ViennaAlthanstrasse 14A‐1090ViennaAustria; ^2^ Stream Biofilm and Ecosystem Research Laboratory, School of Architecture, Civil and Environmental EngineeringÉcole Polytechnique Fédérale de Lausanne (EPFL)CH‐1015LausanneSwitzerland; ^3^ WasserCluster Lunz GmbHDr. Carl Kupelwieser Promenade 53293Lunz am SeeAustria

## Abstract

Streams and rivers transport dissolved organic matter (DOM) from the terrestrial environment to downstream ecosystems. In light of climate and global change it is crucial to understand the temporal dynamics of DOM concentration and composition, and its export fluxes from headwaters to larger downstream ecosystems. We monitored DOM concentration and composition based on a diurnal sampling design for 3 years in an Alpine headwater stream. We found hydrologic variability to control DOM composition and the coupling of DOM dynamics in the streamwater and the hyporheic zone. High‐flow events increased DOM inputs from terrestrial sources (as indicated by the contributions of humic‐ and fulvic‐like fluorescence), while summer baseflow enhanced the autochthonous imprint of DOM. Diurnal and seasonal patterns of DOM composition were likely induced by biological processes linked to temperature and photosynthetic active radiation (PAR). Floods frequently interrupted diurnal and seasonal patterns of DOM, which led to a decoupling of streamwater and hyporheic water DOM composition and delivery of aromatic and humic‐like DOM to the streamwater. Accordingly, DOM export fluxes were largely of terrigenous origin as indicated by optical properties. Our study highlights the relevance of hydrologic and seasonal dynamics for the origin, composition and fluxes of DOM in an Alpine headwater stream.

Streams and rivers are major contributors to global carbon fluxes (Cole et al. 2007; Battin et al. 2009). Headwaters, the smallest and most abundant streams in fluvial networks, are tightly connected with the terrestrial milieu from which they receive dissolved organic matter (DOM) that they transform or export to downstream ecosystems and ultimately to the oceans (Battin et al. 2008). Therefore, headwater streams link the terrestrial and aquatic carbon cycle. Building evidence shows that hydrology drives this biogeochemical link to a large extent. Export budgets of DOM from catchments, DOM composition and its bioavailability to the heterotrophic metabolism in streams are to some extent controlled by precipitation and discharge. For instance, near‐stream soils often rich in organic carbon can act as a DOM source to streams during elevated precipitation and snowmelt (Boyer et al. 1997; Sawyer et al. 2014). During storms, the delivery of DOM from soils can be facilitated by groundwater flowpaths (Sawyer et al. 2014) and even lead to the transient depletion of organic matter in soils (Boyer et al. 1997; Butturini et al. 2006). The combined effects of hydrological connectivity and hydrological flow paths, and the depletion of organic matter reservoirs can lead to significant DOM export fluxes from catchments during storms (Raymond and Saiers 2010; Yoon and Raymond 2012). Using high‐resolution monitoring, Yoon and Raymond (2012) showed that almost 40% of the annual export flux of dissolved organic carbon (DOC) from Esopus Creek occurred during Hurricane Irene within only 5 days. As storms shift DOM sources, its chemical composition in streams may change as well (e.g., Strohmeier et al. 2013; Singh et al. 2014). Furthermore, bioassay experiments have revealed that DOM compounds transported during storms are bioavailable to heterotrophic metabolism (Buffam et al. 2001; McLaughlin and Kaplan 2013). However, reduced residence times and dilution effects due to elevated water level may depress in situ heterotrophic metabolism and, therefore, increase downstream subsidies of labile DOM during storms (Battin et al. 2008). This pattern may reverse when extended baseflow favors fueling of heterotrophic metabolism by in‐stream DOM from benthic algae (Kaplan and Bott 1989), for instance. However, relatively little is known about the DOM dynamics between storms (Vázquez et al. 2012; Wyatt et al. 2014).

The hyporheic zone (i.e., the interface between groundwater and streamwater) is critical for stream ecosystem processes. The hyporheic zone seems particularly important in streams with steep slopes and alluvial geology that facilitate hydrodynamic exchange and hyporheic flow (Boulton et al. 1998; Boano et al. 2014). While the involvement of the hyporheic zone for stream metabolism and nutrient cycling is well recognized (e.g., Schindler and Krabbenhoft 1998; Sobczak and Findlay 2002), the contributions of hyporheic processes to DOM sources and dynamics are poorly understood (Wong and Williams 2010). To assess the contributions of streams to carbon fluxes, we need a better understanding of surface and hyporheic processes affecting DOM sources and fluxes, and of how these may respond to shifts in hydrology and temperature as a consequence of climate change. In fact, alpine ecosystems are particularly susceptible to climate change with predicted changes in precipitation and runoff regime (Barnett et al. 2005; Berghuijs et al. 2014).

The aim of this study was to unravel controls on DOM dynamics and composition in the streamwater and hyporheic zone of an Alpine headwater stream. We monitored diurnal patterns of DOM concentration, absorbance, and fluorescence in the streamwater and the hyporheic zone and computed export fluxes of bulk DOM and its major fluorescent components for over 3 years. We hypothesized that DOM sources and dynamics are differently controlled across the temporal scales of seasonality, day‐night changes, and discrete events such as storms and extended baseflow. We expected storms to increase export fluxes of terrestrial DOM and periods of extended baseflow to contribute autochthonous constituents to the DOM fluxes. Moreover, we expected sources and composition of DOM to differ in streamwater and in the hyporheic zone and that discharge modulated this divergence.

## Methods

### Study site

Oberer Seebach (OSB) is a second‐order stream draining a largely pristine catchment in the eastern Alps (Lunz am See, Austria) of approximately 25 km^2^. *Fraxinus excelsior, Acer pseudoplatanus, Fagus sylvatica, Salix caprea, and Picea abies* dominate the catchment vegetation. Long‐term annual air temperature averaged 6.7°C and annual precipitation averaged 1608 mm. The catchment is characterized by glacial alluvial deposits, which are underlain by a layer of ancient lake sediment and calcareous rock (Battin 1999). OSB streamwater pH and electrical conductivity average 8.3 ± 0.2 and 231.6 ± 17.0 *μ*S cm^−1^, respectively. Nutrient concentrations (P‐PO_4_: <3 *μ*g L^−1^; N‐NH_4_: 4.4 *μ*g L^−1^; N‐NO_3_: 0.57 mg L^−1^) and turbidity (5.4 ± 36.2 NTU) are generally low and the streamwater is typically well oxygenated. Additional key properties of the OSB are summarized in Table 1. The streambed in the study reach is characterized by gravel (median grain size 23.1 mm), high porosity (29%), and a slope of 0.41%, facilitating high hydrological exchange between streamwater and the hyporheic zone (Battin 1999). Snow cover can extend from December until April.

**Table 1:** Environmental data for Oberer Seebach (May 2010 to August 2013). Shown are average values ± standard deviation for spring (20.03–21.06), summer (20.06–22.09), autumn (23.09–21.10), winter (22.10–10.03). Minimum and maximum values are given in parantheses.

	Spring	Summer	Autumn	Winter
Discharge (L s^−1^)	1972 ± 2141	948 ± 1584	609 ± 1216	1079 ± 2019
	(150 ‐ 22,461)	(18 ‐ 16,714)	(94 ‐ 17,674)	(180 ‐ 28,065)
	*n*=1116	*n*=1296	*n*=1080	*n*=1060
Streamwater temperature (°C)	7.13 ± 1.31	8.99 ± 1.28	6.12 ± 1.4	4.77 ± 0.95
	(3.82 ‐ 15.29)	(6.60 ‐ 14.98)	(2.31 ‐ 10)	(1.12 ‐ 8.54)
	*n*=899	*n*=1146	*n*=869	*n*=970
Air temperature (°C)	10.53 ± 6.81	15.76 ± 5.34	3.63 ± 6.68	−1.14 ± 5.77
	(−6.91 ‐ 33.2)	(1.23 ‐ 34.3)	(−20.4 ‐ 22.9)	(−23.2 ‐ 17.9)
	*n*=1253	*n*=1403	*n*=1080	*n*=1060
Streamwater electrical conductivity (μS cm^−1^)	214.5 ± 13.7	230.9 ± 18.2	243.8 ± 8.23	237.1 ± 10.1
	(167 ‐ 238)	(166 ‐ 266)	(209 ‐ 276)	(189 ‐ 290)
	*n*=899	*n*=1146	*n*=869	*n*=970
Streamwater DOC concentration (mg C L^−1^)	1.74 ± 0.33	1.79 ± 0.40	1.56 ± 0.30	1.67 ± 0.37
	(1.11 ‐ 3.87)	(1.11 ‐ 5.43)	(1.16 ‐ 3.38)	(1.13 ‐ 5.25)
	*n*=1055	*n*=1144	*n*=631	*n*=865
Hyporheic DOC concentration (mg C L^−1^)	1.48 ± 0.21	1.55 ± 0.35	1.58 ± 0.35	1.37 ± 0.21
	(1.11 ‐ 2.37)	(0.82 ‐ 2.67)	(1.12 ‐ 3.12)	(1 ‐ 1.90)
	*n*=410	*n*=768	*n*=149	*n*=434
PAR (Watt m^−2^)	154.8 ± 234.5	168.3 ± 246.5	50.7 ± 118.4	50.81 ± 122.1
	(0 ‐ 983)	(0 ‐ 979)	(0 ‐ 603)	(0 ‐ 734)
	*n*=1253	*n*=1402	*n*=1080	*n*=1060

### Hydrology

Streamwater and groundwater levels adjacent to OSB were recorded in 30‐min intervals using pressure transducers (GPRS data logger type 255, HT‐Technik, Germany). We derived stream discharge (L s^−1^) from rating curves (*r* ^2^ = 0.96) based on repeated chloride additions and covering discharges ranging from 80 L s^−1^ to 2520 L s^−1^; higher discharge was extrapolated from this rating curve. Horizontal groundwater discharge (L m^−2^ s^−1^) into and out of OSB was estimated from hydraulic gradients (m m^−1^) between groundwater gauges on both banks of the study reach and from sediment permeability (m s^−1^) (Battin 1999).

### Sampling and sample preparation

We collected streamwater (*n* =3695) and hyporheic (*n* =1761) samples from the same spot in the thalweg of OSB on a diurnal basis (01:30 h, 07:30 h, 13:30 h, and 19:30 h); hyporheic water was sampled from a well installed at 1.5 m below sediment surface. An ISCO autosampler transferred water into clean bottles (soaked in 0.1 N HCl, rinsed with ultraclean MilliQ water), which we then filtered (Whatman GF/F) and stored (4°C in the dark) in borosilicate vials (soaked in 0.1 N HCl, rinsed with MilliQ and combusted at 450°C for 4 h) pending analyses for DOC and DOM absorbance and fluorescence. Streamwater temperature (°C), electrical conductivity (*μ*S cm^−1^), pH and dissolved oxygen (mg L^−1^) were measured with an YSI 600R probe (Ohio, U.S.A.). We also recorded photosynthetic active radiation (PAR) (W m^−2^), air temperature (°C), and precipitation (mm) on site.

### DOC concentration and DOM composition

DOC concentration was determined using a TOC analyzer (GE‐Sievers 900) operated with an inorganic carbon removal unit. We measured DOM absorbance using a UV–VIS spectrophotometer (ShimadzuUV 17000) in 5‐cm cuvettes; ultraclean water (MilliQ) served as blank. Excitation emission matrices (EEMs) were generated using a fluorescence spectrophotometer (Hitachi F‐7000) and 1‐cm quartz cuvettes. Fluorescence intensities were measured at excitation (Ex) wavelengths ranging from 240 nm to 450 nm (5‐nm increments) and emission (Em) wavelengths from 250 nm to 550 nm (2‐nm increments). EEMs were corrected for blanks and the inner filter effect using corresponding absorbance measurements. The Raman peak of ultraclean water (Milli‐Q) was used as reference value to express fluorescence intensities in Raman units. We modeled individual fluorescent components from the EEMs employing parallel factor analysis (PARAFAC) (Stedmon and Bro 2008) using the DOMFluor Toolbox (1.7; containing the N‐Way toolbox, 3.1) (Andersson and Bro 2000). The results of the PARAFAC revealed five fluorescent components. Fluorescence and absorbance parameters used to describe OSB DOM are summarized in Table 2.

**Table 2:** Description of fluorescence and absorbance indices used in the present study to characterize dissolved organic matter in Oberer Seebach. Em = emission wavelength (nm), Ex = excitation wavelength (nm).

	Calculation	Description
SUVA_254_	${SUVA}_{254}=\frac{A_{254}}{DOC}$	The UV absorbance at 254 nm divided by DOC concentration in mg C L^−1^ is indicative of aromaticity (Weishaar et al. 2003)
Slope ratio	$S_{R}=\frac{S_{275‐285}}{S_{350‐400}}$	Ratio of the slopes of the UV wavelength region (275–295 nm) to that of the longer UV wavelength region (350–400 nm); serves as a proxy for the apparent DOM molecular weight (Helms et al. 2008)
Fluorescence index	$FI=\frac{Em\;450}{Em\;500}$ at Ex of 370	The FI serves as indicator of DOM sources (i.e., allochthonous vs. authochthonous) (McKnight 2001)
Humification index	$HIX=\frac{\sum 435‐480}{\sum 300‐445}$ at Ex of 254	Used to characterize humification status of DOM; higher values indicate an increasing degree of humification. (Zsolnay et al. 1999)
Freshness index	$\frac{\beta }{\alpha }=\frac{Em380}{{Em}_{\text{max}}between\;420\;and\;436}$ at Ex of 310	The ratio of Em 380 nm (*β*) to the maximum Em intensity between 420 and 435 nm (*α*) at Ex 310 nm; high *β*/*α* values are commonly associated with autochthonous DOM from recent microbial production (Wilson and Xenopoulos 2008)
C1	Ex 240 (305) nm/ Em 412 nm	Previously reported from forested streams and wetlands; of terrigenous origin (Stedmon and Markager 2005)
C2	Ex 240 (405) nm/ Em 486 nm	Fulvic acid fluorophore group; likely of terrestrial or autochthonous origin and commonly found in freshwaters (Stedmon and Markager 2005; Cory and Kaplan 2012)
C3	Ex 240 (365) nm/ Em 458 nm	Fulvic acid fluorophore group; likely of terrestrial or autochthonous origin and commonly found in freshwaters (Stedmon and Markager 2005; Cory and Kaplan 2012)
C4	Ex 240 (280) nm/ Em 338 nm	Tryptophan‐like fluorescence (Coble 1996)
C5	Ex 270 nm/ Em 300 nm	Tyrosine‐like fluorescence (Coble 1996)

### Flux calculations

We estimated average annual discharge and DOC export fluxes from OSB according to Raymond and Saiers (2010). Briefly, we binned DOC concentrations by averaged daily discharge where the highest discharge bin contained DOC concentrations equated to discharge higher than 58 mm d^−1^ (that is, the lower bound of the highest bin). The bounds of the remaining bins were computed from an exponential function. We estimated the annual discharge associated with each bin by multiplying the binned discharge by the fraction of days at which the corresponding discharge was measured. Bin‐averaged DOC concentrations (mg L^−1^) were then described as a power law function of discharge (Q, mm d^−1^), which allows calculating DOC concentrations for all discharges, including discharges for which DOC measurements were unavailable (Raymond and Saiers 2010). The same procedure was repeated for the rising $(DOC=1.19+0.29\;Q^{0.403}$, *r* ^2^=0.98, *p* < 0.001) and falling ( $DOC=1.26+0.24\;Q^{0.396}$, *r* ^2^=0.94, *p* < 0.001) limb of the hydrograph to determine DOC export fluxes for both situations. We used bin‐averaged values for discharge and DOC concentration to calculate DOC export fluxes at baseflow. We also calculated export fluxes of DOC and fluorescent components (using absolute values; Raman Units [R.U.]) for the whole study period and for each month individually using LOADest (Runkel et al. 2004). These fluxes (R.U. m^−3^) were then normalized to catchment area (m^2^) to derive yields of optical components (R.U. m^−1^ yr^−1^). Furthermore, we used the SUVA_254_ (m^−1^) and discharge (m^−3^ day^−1^) to compute the flux (m^−2^ yr^−1^) and yield (yr^−1^) of aromatic DOM. Individual models were validated using adjusted maximum likelihood estimations, residuals data, and the serial correlation of residuals according to Dornblaser and Striegl (2009).

### Data analysis

Principal component analysis (PCA) based on DOM absorbance and fluorescence measurements was used to explore the variability of DOM composition in the streamwater and the hyporheic zone. To assess effects of discharge on DOM composition, we calculated linear regression models on the relationship between the scores of the principal component 1 and discharge. Furthermore, we computed a Procrustes rotation (“procrustes” function in R, R Core Team 2015) of the streamwater and the hyporheic water PCA for low, high, and intermediate discharge to evaluate the effect of discharge on the coupling between streamwater and hyporheic water optical properties at different flow conditions. To further analyze the effect of environmental variables (e.g., discharge, streamwater temperature) on the coupling of DOM optical properties in both waters, we computed a canonical correlation analysis. The coupling of the DOM optical properties was computed as the relative difference between values of streamwater and hyporheic water DOM optics (ΔDOM). Positive values indicate higher absorbance and fluorescence in the streamwater than in the hyporheic zone. We calculated canonical loadings to analyze the strength of the relationship between the DOM optical indices and the canonical variates (based on the dataset containing Q, PAR and streamwater temperature and the dataset containing DOM optical properties). We then computed the significance of each canonical correlation using permutations with the “CCP” package (Menzel et al. 2009; R Core Team 2015). We analyzed diurnal variation in DOC concentration (dDOC) using linear models selected according to AIC criteria in comparison to the simplest model. The results of these models revealed differences to the simplest model of ΔAIC = 28 and ΔAIC = 4 for streamwater and hyporheic diurnal DOC concentrations, respectively. We explored the effect of discharge and streamwater temperature on streamwater DOM concentration and composition using general additive models (GAM) with a residual temporal correlation structure (auto‐regressive model of order 1). GAMs were fitted the “mgcv” package in R (R Core Team 2015; Wood et al. 2015).

## Results

### OSB hydrology

**Figure 1:**
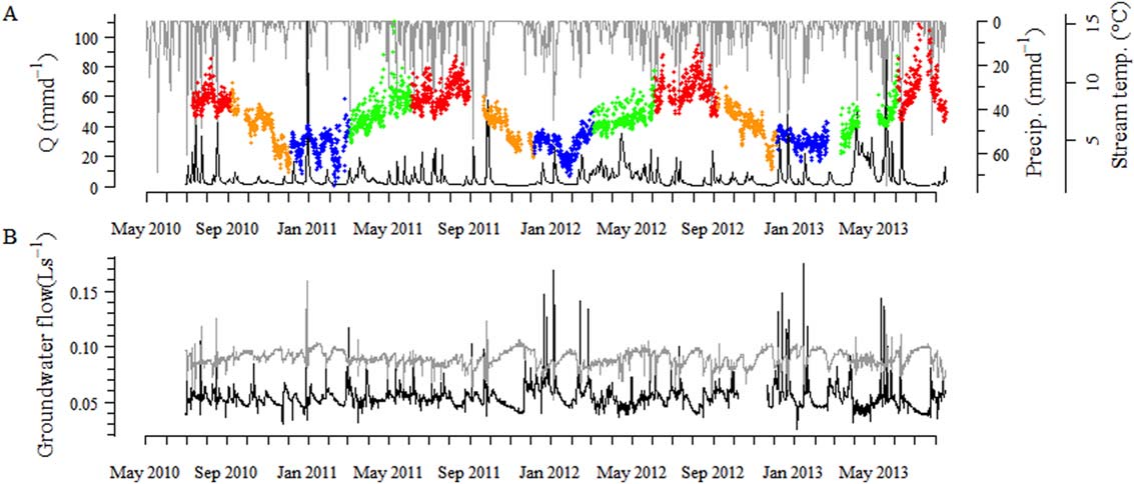
(A) Daily discharge (black), daily precipitation (gray), and streamwater temperature (color indicates seasons: spring [green], summer [red], autumn [orange], winter [blue]); (B) groundwater inflow (black) and outflow (gray) during the study period in Oberer Seebach, Austria.

Distinct snowmelt events with a mean peak discharge of 28 mm day^−1^, extended baseflow (0.45 mm day^−1^ to 1.47 mm day^−1^) in summer interrupted by storms (up to 60 mm day^−1^) characterized the hydrological regime of OSB (Fig. 1A). Overall discharge of OSB during our study period averaged 1152 L s^−1^ (equivalent to a specific discharge of 5.7 mm d^−1^) with peak values of 5575 ± 3352 L s^−1^. Based on a flow‐duration curve of average daily discharge, we defined discharge lower than 75% of the time (< 306 L s^−1^) as baseflow. Similarly, high discharge was defined as the discharge of the highest 10% of the days (discharge > 2939 L s^−1^). Discharge between these 10% and 75% duration limits (306 L s^−1^ to 2939 L s^−1^) were considered as intermediate. These thresholds agree approximately with previous estimates of median baseflow (289 L s^−1^) and threshold values of bed scouring discharge (3400 L s^−1^) for OSB (Peter et al. 2014).

The discharge regime was linked to the dynamics of groundwater entering the study reach from the left hill slope (Fig. 1B). Overall the OSB study reach was losing water through the right bank with average outflowing fluxes of 9.4 ± 0.4, 8.7 ± 0.6, and 8.5 ± 1.3 × 10^−2^ L s^−1^ m^−2^ and average inflowing fluxes of 4.8 ± 0.6, 5.6 ± 0.9, and 5.5 ± 1.6 × 10^−2^ L s^−1^ m^−2^ during low, intermediate, and high discharge, respectively.

### DOC concentration and DOM composition

The annual average DOC concentration in OSB streamwater was 1.71 ± 0.35 mg L^−1^ with maximum concentrations of 2.18 ± 0.52 mg L^−1^ during storms (Table 1). DOC concentration in the hyporheic zone ranged from 0.82 mg L^−1^ to 3.12 mg L^−1^ and its average (1.5 mg L^−1^ ± 0.3 mg L^−1^) was significantly lower (*t*‐test, *p* < 0.001, *n* = 1746) than in the streamwater.

DOC concentration in the streamwater was related to discharge (*r* ^2^ = 0.43, *n*= 3495, *p* < 0.001); hyporheic DOC concentration was related to streamwater DOC concentration and discharge (multiple linear regression, adjusted *r* ^2^ = 0.68, *n* = 5238, *p* < 0.001).

**Figure 2:**
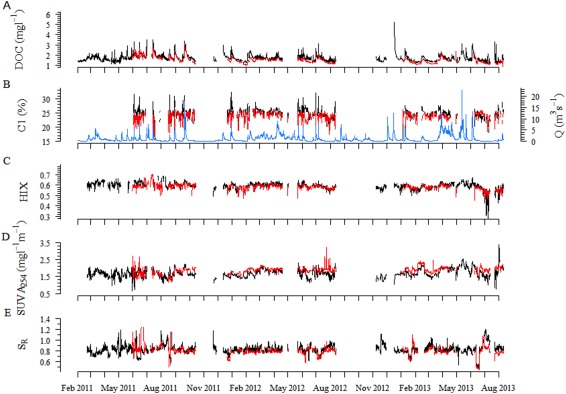
(A) DOC concentration (mg C L^−1^), (B) stream discharge (Q, m^3^ s^−1^) and the fluorescent component C1 (%) modeled by parallel factor analysis (PARAFAC), (C) HIX, (D) SUVA_254_ (mg L^−1^ m^−1^), and (E) the slope ratio (*S*_R_) during the study period.

**Figure 3:**
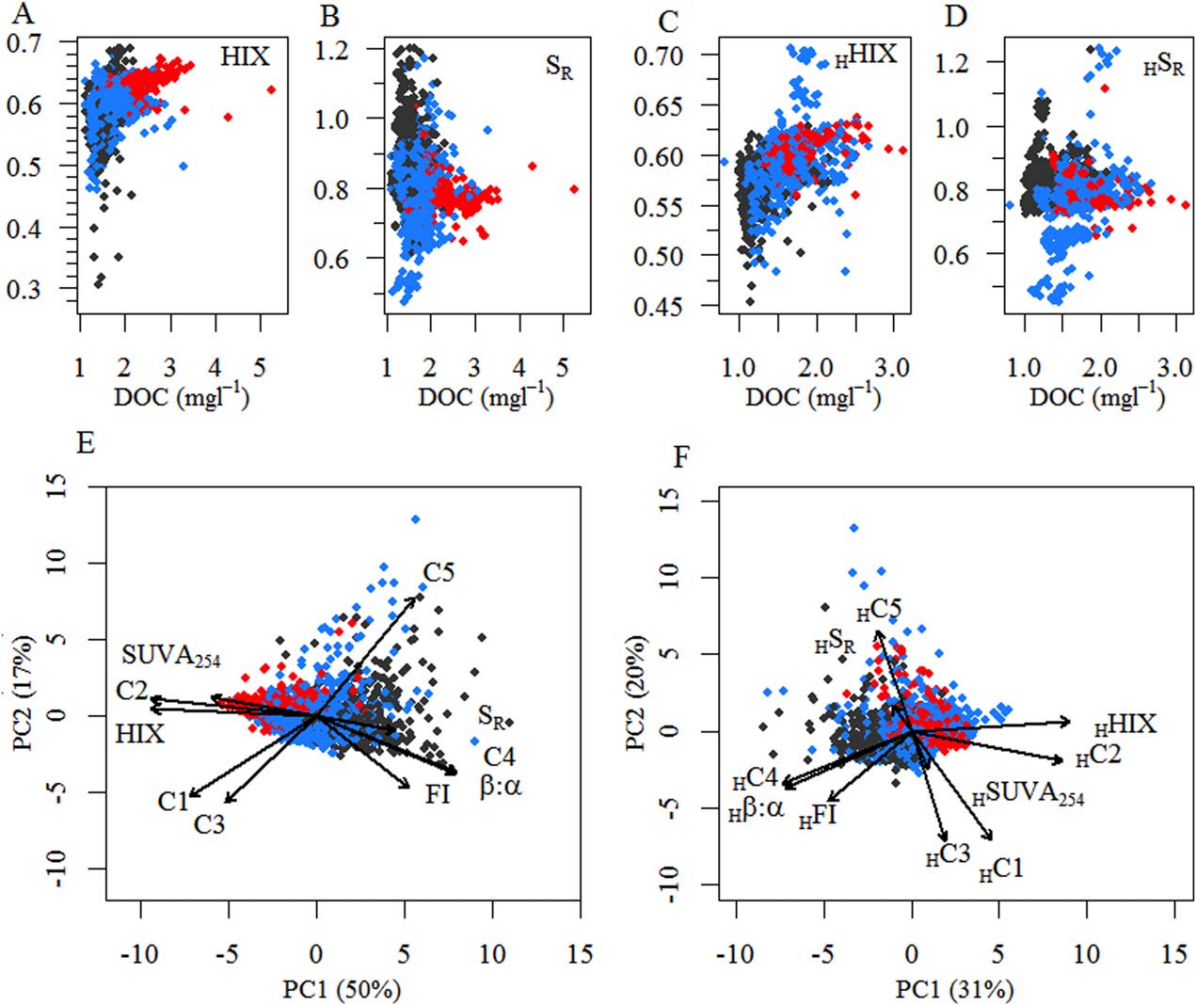
Dissolved organic matter composition in Oberer Seebach. Shown are representative relationships of the dissolved organic carbon (DOC) concentration and representative measures derived from absorbance and fluorescence measurements in the (A, B) streamwater and the (C, D) hyporheic zone: humification index (HIX) and the slope ratio (*S*_R_). Principal component analysis (PCA) based on absorbance and fluorescence measurements in the (E) streamwater, and the (F) hyporheic zone: Humic‐ like components (C1–C3), protein‐like components (C4 and C5), specific absorption at 254 nm (SUVA_254_), humification index (HIX), fluorescence index (FI), freshness index (*β*/*α*), slope ratio (*S*_R_) (*see* Methods). Arrows are based on PCA structural coefficients. Black symbols indicate baseflow (< 306 L s^−1^), blue and red symbols indicate intermediate flow (306–2939 L s^−1^) and high flow (> 2939 L s^−1^), respectively.

Streamwater and hyporheic DOC concentrations and DOM optical properties varied considerably during the study period (Fig. 2). For instance, the coefficient of variation (CV) for streamwater *S*_R_ increased from 5.39 at high discharge to 10.39 at baseflow. Similarly, the CV of HIX changed from 3.08 to 6.63. Streamwater DOM composition generally exhibited a greater variability than hyporheic water DOM. The CV of streamwater SUVA_254_ was 14.94 and 10.42 for the hyporheic zone, and 5.48 and 4.58 for streamwater and hyporheic HIX, respectively. Elevated streamwater DOC concentrations (> 2.2 mg C L^−1^) were typically related to more terrigenous DOM as indicated by elevated high HIX (Spearman Correlation, *r* = 0.29, *p* < 0.001, *n* = 4880) and reduced *S*_R_ (Spearman Correlation, *r* = 0.57, *p* < 0.001, *n* = 5154) at high discharge (> 2939 L s^−1^)—a pattern that was not as pronounced in the hyporheic zone (Fig. 3A–D). Relating the scores of the principal component 1 to discharge, confirmed discharge as a control on streamwater DOM composition (*r* ^2^ = 0.29, *p* < 0.001, *n* = 2208) but to a lesser extent on hyporheic DOM composition (*r* ^2^ = 0.1, *p* < 0.001, *n* = 1349; Fig. 3E,F). Hyporheic DOM optical properties followed a similar pattern; however, variability was most pronounced at intermediate discharge (Fig. 3C,D). For instance, the CV of *S*_R_ was highest (CV = 11.72) at intermediate flow but decreased at both low (CV = 7.74) and high flow (CV = 5.68). The results of the procrustes rotation of the streamwater and the hyporheic water PCA revealed a strong coupling of streamwater and hyporheic DOM pattern at baseflow conditions (Procrustes Sum of Squares 0.789, correlation 0.459, *p* = 0.001), which decreased as Q increases (intermediate Q: Procrustes Sum of Squares 0.709, correlation 0.054, *p* = 0.001; high Q: Procrustes Sum of Squares 0.784, correlation 0.046, *p* = 0.001).

Elevated discharge induced a terrigenous imprint on streamwater and hyporheic DOM as indicated by elevated SUVA_254_ and contributions of C1, C2, and C3. This DOM pool was further characterized by elevated HIX and lower *S*_R_ values. At baseflow, values of *β*/*α*, FI, and *S*_R_ increased while SUVA_254_ decreased, which suggests autochthonous deliveries to the DOM pool. This pattern was further supported by elevated contributions of tryptophan‐like (C4) and tyrosine‐like fluorescence (C5).

**Figure 4:**
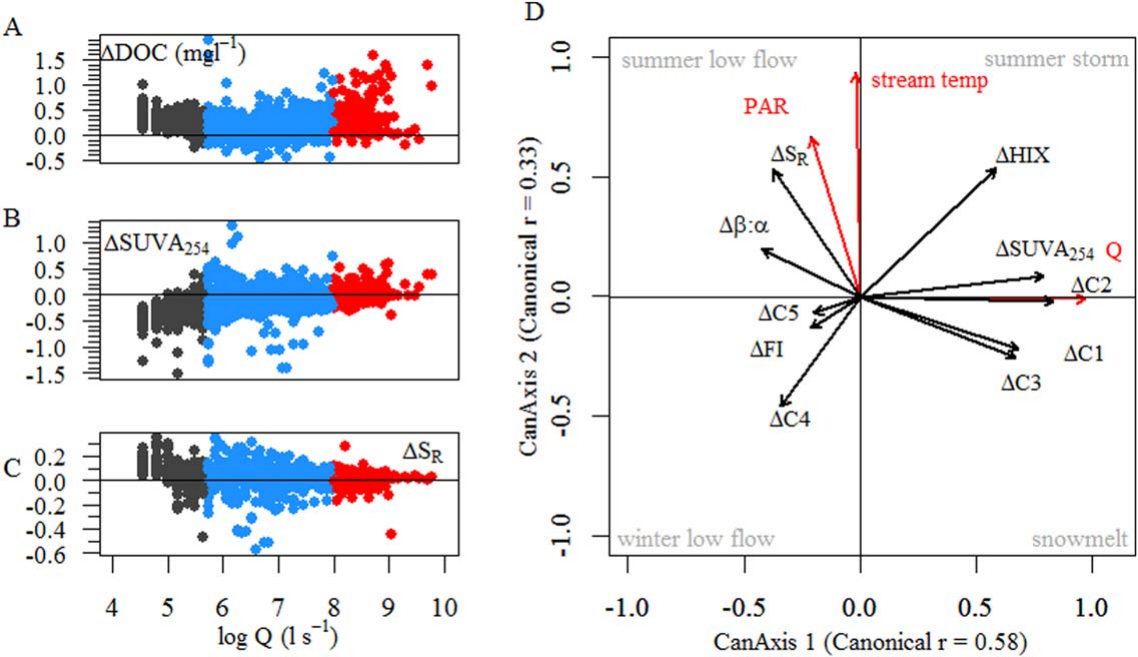
Representative patterns of DOM optical measures in the streamwater and the hyporheic zone (hyporheic water optical measures were subtracted from streamwater optical measures at the same time points): (A) dissolved organic carbon (ΔDOC), (B) specific UV absorbance at 254 nm (ΔSUVA_254_), and (C) slope ratio (ΔS_R_). Color coded by discharge (Q) bins. (D) Canonical correlation analysis based on environmental parameters and DOM composition in the streamwater and the hyporheic zone in Oberer Seebach (*n* = 1040) indicates the main drivers of the coupling between streamwater and hyporheic water DOM: Shown are streamwater temperature (stream temp), discharge (Q), photosynthetic active radiation (PAR) and DOM optical measures (streamwater minus hyporheic water) derived from absorbance and fluorescence measurements (for more information *see* text). Arrows represent standardized canonical coefficients. Annotations in the corners (summer low flow, summer storm, winter low flow, winter storm) indicate typical events corresponding to the ensemble of Q, PAR, and streamwater temperature influencing DOM composition.

The temporal dynamics of DOM composition differed in the streamwater and hyporheic zone. For instance, DOC concentration and *S*_R_ were higher in the streamwater than in the hyporheic zone at low flow (207 ± 59 L s^−1^), whereas this pattern inverted for SUVA_254_ (Fig. 4A–C). These trends became weaker at intermediate discharge (887 ± 615 L s^−1^) except a clear overall increase of DOC concentration at high discharge (5195 ± 2831 L s^−1^). Canonical correlation analysis (CCA) based on the differences of streamwater and hyporheic DOM composition (ΔDOM) confirmed the role of discharge as a driver of the difference of DOM composition in the streamwater and the hyporheic zone, and further suggested PAR and streamwater temperature as predictors (canonical *r* = 0.67, *p* < 0.001, *n* = 1040; Fig. 4D; Table 3, 4). CCA suggests that the contributions from autochthonous sources (as indicated by elevated *β*/*α* and *S*_R_ values) were higher in the streamwater than in the hyporheic zone at baseflow. For instance, during summer baseflow (lower discharge and high PAR in the CCA), streamwater *β*/*α* and *S*_R_ increased with increasing streamwater temperature and PAR while hyporheic SUVA_254_ was highest during baseflow. During snowmelt (higher discharge and low temperatures) and summer storms (higher temperature) this pattern reversed with increased contributions of the humic‐like components (C1, C2, and C3), HIX, and SUVA_254_ to streamwater DOM compared to hyporheic water.

**Table 3:** Canonical correlation analysis based on discharge (Q), streamwater temperature and photosynthetic active radiation (PAR) and dissolved organic matter properties derived from absorbance and fluorescence measurements from the Oberer Seebach; hyporheic DOM optical measures were subtracted from streamwater DOM optical measures (*n*=1040). Optical properties included HIX, FI, *β*/*α*, *S*_R_, SUVA_254_, humic‐like components (C1, C2,C3), protein‐like components (C4, C5). Shown are tests of canonical dimensions. The significance of each canonical correlation was computed by permutation.

Dimension	Canonical *r*	*p*‐value
1	0.67	< 0.001
2	0.33	< 0.001
3	0.28	< 0.001

**Table 4:** Canonical correlation analysis based on discharge (Q), streamwater temperature and photosynthetic active radiation (PAR) and dissolved organic matter properties derived from absorbance and fluorescence measurements from the Oberer Seebach (*see* Table 2). Shown are canonical loadings for the first two canonical dimensions.

Dimension	1	2
Q	0.64	−0.0004
Streamwater temperature	−0.14	0.22
PAR	−0.01	0.31
ΔHIX	0.39	0.18
ΔFI	−0.13	−0.02
Δ*β*/*α*	−0.28	0.07
Δ*S*_R_	−0.25	0.18
ΔSUVA_254_	0.52	0.03
ΔC1	0.45	−0.07
ΔC2	0.55	−0.01
ΔC3	0.45	−0.09
ΔC4	−0.23	−0.15
ΔC5	−0.14	−0.04

### Diurnal patterns of DOM composition

**Figure 5:**
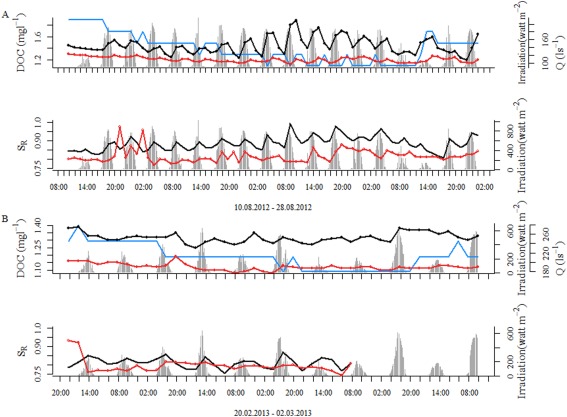
Representative patterns of diurnal variation of streamwater (black) and hyporheic water (red) DOC concentration (mg L^−1^) and slope ratio (*S*_R_) in (A) summer and (B) winter. Diurnal amplitudes were more pronounced in summer than in winter. Discharge (Q) is indicated in blue and photosynthetic active radiation (PAR) in gray.

At baseflow, streamwater and hyporheic DOC concentrations followed diurnal patterns, which were most pronounced during summer but less during winter (Fig. 5). Diurnal variations of streamwater DOC concentration ranged from 0.01 mg C L^−1^ to 1.25 mg C L^−1^, averaging 0.22 ± 0.16 mg C L^−1^ in summer and 0.08 ± 0.04 mg C L^−1^ in winter, respectively. Maximum daily DOC concentrations occurred around 19:30 h as PAR decreased. To explore the variation and potential drivers of the diurnal amplitude of streamwater DOC concentration (dDOC) at baseflow, we proposed the following linear model:

(1) $$dDOC=b_{0}*PAR+b_{1}*T_{Streamwater}+b_{2}*time\;after\;storm.$$

This model was based on the assumption that PAR and streamwater temperature drive in‐stream biological activity, which may build up as a function of the time between storms. We further assumed that this biological activity drives to some extent the observed diurnal patterns. We found PAR (beta = 0.25, *p* < 0.001, sum of squares = 0.16), streamwater temperature (beta = 0.28, *p* < 0.001, sum of squares = 0.37) and the time since the last storm event (beta = 0.20, *p* < 0.01, sum of squares = 0.08) to explain 45% of the daily amplitude of ΔDOC (*n* = 149). This model was selected over the simpler model based on streamwater temperature only (ΔAIC = 28).

In the hyporheic zone, diurnal amplitudes of DOC concentration (dDOC_H_; 0.01–0.26 mg C L^−1^) were less pronounced than in the streamwater. The maximum amplitude of dDOC_H_ occurred between 19:30 h and 01:30 h. Hydrodynamic exchange between streamwater and the hyporheic zone in OSB is controlled by discharge (Battin 1999), and we, therefore, proposed the following linear model to explore variations in ΔDOC_H_

(2) $$\log d{DOC}_{H}=b_{0}*log\;dDOC+b_{1}*\log Q_{baseflow}$$

We found that dDOC (beta = 0.69, *p* < 0.001, sum of squares = 18.45) and discharge at baseflow (*Q*_baseflow_; beta = 0.66, *p* < 0.05, sum of squares = 2.58) explained 31% of the variance in ΔDOC_H_ at baseflow (*n* = 119).

Besides the diurnal patterns of DOC concentration, we also found that *S*_R_ changed on a diurnal basis with peak values at 13:30 h and minimal values at night (Fig. 3). Diurnal variations of *S*_R_ occurred predominantly during summer in streamwater (0.07 ± 0.05), but were less pronounced in the hyporheic zone (0.05 ± 0.04). Also, variation of *S*_R_ was small during winter in both the streamwater and the hyporheic zone as compared to the summer. Interestingly, the other optical properties did not exhibit clear diurnal patterns.

### Seasonal patterns of DOM composition

Streamwater DOM composition varied among seasons. For instance, baseflow peak *S*_R_ was 0.9 ± 0.09 in summer but only 0.8 ± 0.04 in winter. HIX varied broadly in summer (0.28 to 0.74, CV = 7.48) but less in winter (0.50 to 0.64, CV = 4.27). The protein‐like component C4 displayed maximum values (up to 30%) in summer and decreased in autumn (8%). As revealed by the results of the GAM analyses (Table 5), streamwater DOC concentration, *β*/*α* and tryptophan‐like fluorescence were positively related to streamwater temperature. Tryptophan‐like fluorescence and *β*/*α* were inversely related to discharge (Table 5). Values of *β*/*α* and *S*_R_ were elevated in summer at low to intermediate discharge. In contrast, the humic‐like component C1, fulvic‐like components C2 and C3, and HIX were positively related to discharge (Table 5).

**Table 5:** General additive models (GAM) including a residual temporal correlation structure (auto‐regressive model of order 1) of discharge and streamwater temperature and the individual fluorescent components (in %) and DOM optical measures derived from fluorescence and absorbance measurements: dissolved organic carbon (DOC), humification index (HIX), fluorescence index (FI), freshness index (*β*/*α*), slope ratio (*S*_R_), specific UV absorbance at 254 nm (SUVA_254_), humic‐ like components (C1–C3), protein‐like components (C4 and C5). Shown are standardized regression coefficients (*β*), *t*‐values, estimated degree of freedom (edf) and the deviance explained (%).

		Discharge			Streamwater temperature				
	Intercept	*β*	*t*‐value	*p*‐value	*β*	*t*‐value	*p*‐value	edf	Deviance explained (%)
DOC	1.45	1.15 × 10^−4^	50.85	*p*<0.001	1.59 × 10^−2^	4.13	*p*<0.001	8.64	54
HIX	0.62	5.29 × 10^−6^	13.46	*p*<0.001	−3.07 × 10^−3^	−4.84	*p*<0.001	8.99	49
FI	1.38	−5.65 × 10^−6^	−12.66	*p*<0.001	−9.25 × 10^−4^	−1.3	*p*=0.020	8.83	45.8
*β*/*α*	0.63	−6.07 × 10^−6^	−16.57	*p*<0.001	4.90 × 10^−3^	8.3	*p*<0.001	8.98	46.3
*S*_R_	0.64	−4.21 × 10^−6^	−4.95	*p*<0.001	2.51 × 10^−2^	16.45	*p*<0.001	8.80	17.3
SUVA_254_	2.08	4.97 × 10^−5^	24.44	*p*<0.001	−6.28 × 10^−2^	−17.07	*p*<0.001	8.93	45.9
C1	25.50	3.98 × 10^−4^	12.68	*p*<0.001	−1.52 × 10^−1^	−3.39	*p*<0.001	8.88	28.4
C2	9.89	3.45 × 10^−4^	23.64	*p*<0.001	−1.27 × 10^−1^	−5.19	*p*<0.001	8.92	61.3
C3	9.93	2.06 × 10^−4^	17.48	*p*<0.001	−1.43 × 10^−1^	−7.35	*p*<0.001	8.47	45.6
C4	3.78	−2.03 × 10^−4^	−10.38	*p*<0.001	2.98 × 10^−1^	9.04	*p*<0.001	8.89	49.8
C5	1.91	−1.71 × 10^−4^	−4.03	*p*<0.001	5.21 × 10^−1^	0.74	*p*=0.46	8.58	9.82

### DOM export fluxes

Binning discharge (Raymond and Saiers 2010) and LOADest (Runkel et al. 2004) yielded comparable water fluxes of 2002 mm y^−1^ and 2028 mm y^−1^, and nearly identical DOC yields of 4.00 g m^−2^ y^−1^ and 4.03 ± 0.02 g m^−2^ yr^−1^, respectively. These DOC yields were also identical with the sum (4.01 g m^−2^ y^−1^) of DOC export fluxes calculated separately for baseflow, high, and intermediate discharge. The yield of aromatic DOM (*a*254) was estimated at 24.71 ± 0.14 yr^−1^, while yields of the C1, C2, and C3 components (all humic‐like) averaged 0.91 ± 0.005 R.U m yr^−1^, 0.38 ± 0.003 R.U m yr^−1^ and 0.35 ± 0.002 R.U m yr^−1^, respectively (Table 6). The export fluxes of the protein‐like components, C4 and C5, were consistently lower with 0.17 ± 0.003 R.U m yr^−1^ and 0.05 ± 0.002 R.U m yr^−1^, respectively, than the fluxes of the humic‐like components. The humic‐like components C1 (*r* ^2^ = 0.62, *n* = 2639, *p* < 0.001), C2 (*r* ^2^ = 0.66, *n* = 2639, *p* < 0.001) and C3 (*r* ^2^ = 0.62, *n* = 2639, *p* < 0.001) were related to overall DOC concentration; however the variation of the protein‐like components was not related to DOC concentration (*p* < 0.05). We found 66% of DOC and 59–65% of chromophoric DOM (as depicted by the fluorescent components) to be exported in spring and summer

**Table 6:** Loads and export fluxes of DOC (including standard error of prediction calculated for the modeled flow period), the fluorescent components (C1‐C5 in R.U from PARAFAC analysis) and the absorbance at 440 nm (*a*254).

	Load	Flux
DOC	7.27 × 10^7^ ± 4.33 × 10^5^ g yr^−1^	4.03 ± 0.02 g m^−2^ yr^−1^
*a*254	4.46 × 10^8^ ± 2.59 × 10^6^ m^2^ yr^−1^	24.71 ± 0.14 yr^−1^
C1	1.64 × 10^7^ ± 8.43 × 10^4^ R.U m^3^ yr^−1^	0.91 ± 0.005 R.U m yr^−1^
C2	6.84 × 10^6^ ± 5.11 × 10^4^ R.U m^3^ yr^−1^	0.38 ± 0.003 R.U m yr^−1^
C3	6.25 × 10^6^ ± 3.61 × 10^4^ R.U m^3^ yr^−1^	0.35 ± 0.002 R.U m yr^−1^
C4	3.09 × 10^6^ ± 5.15 × 10^4^ R.U m^3^ yr^−1^	0.17 ± 0.003 R.U m yr^−1^
C5	9.58 × 10^5^ ± 3.58 × 10^4^ R.U m^3^ yr^−1^	0.05 ± 0.002 R.U m yr^−1^

## Discussion

This study emphasizes the role of hydrology, seasonal, and diurnal variability for DOM composition and export from an Alpine stream. Our findings on DOM source partitioning with varying discharge and in‐stream biological processes expand current knowledge on carbon dynamics in stream ecosystems (e.g., Raymond and Saiers 2010; Inamdar et al. 2012; Singh et al. 2014). Our findings also shed new light on the relevance of the hyporheic zone as a contributor to stream DOM dynamics.

### Drivers of DOM source and composition

Our optical measurements suggested that DOM in streamwater and hyporheic water was to a large extent of terrestrial origin throughout the year. This conclusion was supported by elevated values of humic‐like (C1) and fulvic‐like components (C2, C3), elevated aromaticity (as SUVA_254_) and by FI values. Snowmelt and summer storms mobilized terrestrial DOM likely originating from humic‐rich riparian soils in the floodplains adjacent to OSB. These findings were consistent with reports from other streams and support the notion of large lateral carbon fluxes entering headwater streams (e.g., Hornberger et al. 1994; Lambert et al. 2013). In addition to this dynamic baseline of allochthonous DOM, autochthonous sources contributed to the DOM composition depending on discharge and season. This observation was essentially supported by the diurnal and seasonal variation of apparent molecular weight (*S*_R_), aromaticity (SUVA_254_), freshness (*α*/*β*), and by results from the PARAFAC.

Hydrology shaped DOM composition and dynamics in the streamwater and hyporheic zone of OSB, permitting seasonal and diurnal patterns at baseflow. The marked seasonal and diurnal variations of apparently autochthonous DOM evoked biological processes linked to PAR and temperature, which were likely coupled yet may act at different temporal scales. While streamwater temperature closely reflected seasonal conditions in OSB (Fig. 1), diurnal patterns were potentially driven by PAR.

Elevated PAR and low discharge augmented the contributions of freshly produced compounds with lower molecular weight to the DOM pool. It was reasonable to assume that benthic biofilms were major sources of this DOM as, for instance algae exude low‐molecular weight compounds (Kaplan and Bott 1989) and bacteria produce fluorescent DOM (Guillemette and del Giorgio 2012). Less pronounced temporal variations of this autochthonous DOM in the hyporheic zone than in the streamwater corroborated the notion of benthic algae as a prime source of this DOM to streamwater. This observation was in agreement with earlier findings in OSB showing that the downward mixing (i.e., downwelling) of streamwater into the hyporheic zone was moderate at baseflow (Battin 1999). In contrast, during baseflow shallow groundwater that entered the OSB streambed may have entrained aromatic DOM rich in humics and fulvics from riparian soils. These terrestrial DOM deliveries varied less in time than the production of autochthonous DOM because groundwater recharged the streambed continuously throughout the year; this continuous allochthonous input of DOM may have, therefore, blurred autochthonous signatures in the hyporheic zone.

During summer baseflow, patterns of diurnal variations in DOC concentration and DOM apparent molecular weight were marked in the streamwater but not in the hyporheic zone. These diurnal patterns coincided with PAR dynamics. Interestingly, apparent molecular weight decreased with PAR, while DOC concentrations peaked as daily PAR decreased. This suggests that biological activity of benthic biofilms, including algae and their exudates, caused these diurnal fluctuations. Further evidence for these in‐stream sources may be derived from the fact that the day‐night oscillations of streamwater DOC concentration increased with the time elapsed since the last storm event. Benthic biomass accumulates as baseflow extends, thereby producing stronger signatures of autochthonous DOM when photosynthesis peaks. Storms may then disrupt this oscillating DOM patterns by scouring the streambed and collapsing primary production (Uehlinger 2006; Peter et al. 2014)—a pattern consistent with our observations from OSB. We cannot rule out, however, a concurrent effect of photooxidation that was also shown to increase *S*_R_ values in OSB (Fasching and Battin 2011). It could be argued that photooxidation of high‐molecular‐weight DOM from terrestrial sources can produce low‐molecular‐weight DOM (Kragh et al. 2008) and thereby increasing *S*_R_ values. Photooxidation rather than biological activity would explain why we did not observe diurnal patterns in any other optical DOM descriptor. However, this argument would be neutralized by the fact that DOC concentration increased as well.

Our study corroborates earlier reports on diurnal variations of DOM in a larger river (San Joaquin River, U.S.A.), which were attributed to both biological and photochemical processes (Spencer et al. 2007). At the same time our data on hyporheic DOM expand the current picture of diurnal DOM patterns in streams. Based on earlier work on hydrodynamic exchange in OSB (Battin 1999), we suggest that the connectivity between benthic and hyporheic processes was primarily controlled by discharge and that this connectivity also imparted the various diurnal dynamics on the DOM in the streamwater and the hyporheic zone. At baseflow both systems were largely decoupled and diurnal autochthonous DOM patterns evolved in the streamwater but less in the hyporheic zone. As discharge increased more water entered the hyporheic zone and the benthic and hyporheic systems became more strongly coupled. At the same time, however, physical disturbance of the benthic zone and abrasion of benthic biofilms along with simple dilution effects, reduced signatures of benthic‐derived DOM in the hyporheic zone.

Seasonal dynamics of discharge, including summer droughts and snowmelt, can further shape DOM composition and concentration (Laudon et al. 2004; Singh et al. 2014). In OSB, seasonal changes in streamwater DOM composition may have resulted from shifts in sources potentially ranging from soils, leaf litter, groundwater, and from in‐stream primary production. Our results suggest, for instance, that during the spring freshet terrigenous DOM was flushed into OSB, while baseflow in winter favored autochthonous DOM production.

We did not find clear seasonal patterns of hyporheic DOM composition as others did (Wong and Williams 2010). Instead, our results indicated that hyporheic DOM was a composite of aromatic DOM from riparian soils that entered the hyporheic zone along subsurface flow paths. In the hyporheic zone, these terrestrial deliveries mixed with DOM downwelling from the streamwater, which depended on discharge. We suggest that the increased variability of hyporheic DOM composition resulted from the mixing of these two end members, which was most pronounced at intermediate discharge. This mixing translated into reduced DOM variability at elevated discharges when allochthonous rather than autochthonous DOM entered the hyporheic zone from the streamwater. Although DOM composition in the streamwater exhibited higher variability than hyporheic DOM composition it followed clear seasonal trends. Similarly Singh et al. (2014) found pronounced seasonality in streamwater DOM composition, yet this was muted or absent in groundwater and during baseflow conditions. However, in OSB, seasonal differences were pronounced at baseflow conditions. For instance, diurnal variations of apparent molecular weight (*S*_R_), indicating photo‐oxidation, and elevated freshness (*β*/*α*) were dominant during summer baseflow, while microbially derived DOM, as indicated by tryptophan‐like fluorescence (C4) and higher FI values (McKnight et al. 2001; Osburn et al. 2012) imprinted the DOM during baseflow in winter. These results indicated that different processes in summer and winter may have contributed to the production of autochthonous DOM. Primary production and photo‐oxidation may have been important in summer, while microbial degradation may have dominated in winter. These processes likely provided labile DOM to the streamwater during baseflow.

Storm events interrupted seasonal and diurnal patterns of DOM composition and diverged its dynamics in the streamwater and the hyporheic zone. Storms likely changed the DOM deliveries from shallow groundwater into OSB at baseflow (Battin 1999) to deliveries from top soils and A‐horizons via surface runoff and shallow subsurface flow. This conjecture is supported by the observation that summer storms increased the contributions of DOM with high humification, aromaticity, and lower FI. Similar shifts in DOM composition were also observed in other streams interpreted as a shift in DOM source from mineral soils to top soils enriched in aromatic substances and lignin (Vidon et al. 2008; Inamdar et al. 2011, 2012). In OSB, storms further depressed the temporal variability of autochthonous DOM constituents, such as protein‐like fluorescence and *S*_R_. We propose that in OSB storms may have entrained aromatics from the surface water and groundwater into the hyporheic zone, thereby turning over hyporheic DOM, which may be relevant for hyporheic biogeochemistry (Sawyer et al. 2014). Collectively, our findings suggest that hydrology and both seasonal and diurnal controls shaped DOM composition in the streamwater, and that hydrodynamics determined DOM composition in the hyporheic zone on top of the allochthonous baseline.

### DOM export fluxes

The DOC flux from OSB was within the range of fluxes from small, forested watersheds in the northeastern U.S.A. (0.5–5.7 g m^−2^ yr^−1^; Raymond and Saiers 2010). The *a*254 yield from the OSB was relatively low compared to large rivers (Spencer et al. 2013), which may be attributed to difference in catchment size. However, the areal flux of *a*254 from the OSB (24.71 ± 0.14 yr^−1^) was bracketed by the fluxes from large rivers (0.038–67.53 yr^−1^) (Spencer et al. 2013). Clearly, discharge rather than concentration was the more important control on DOM export fluxes from OSB, as fluxes largely mirrored variations in discharge. This resulted in relatively high humic and fulvic export fluxes throughout the year. These fluxes peaked in spring and summer, which was attributable to snowmelt and summer storms. Hydrologic events have previously been shown to impact DOC fluxes and induce a hysteretic DOC response to snowmelt (Boyer et al. 1997; Laudon et al. 2004; Pellerin et al. 2012) and storm events (Hornberger et al. 1994; Butturini et al. 2006). We found that streamwater DOC concentrations increased more rapidly with increasing discharge during the rising limb of the hydrograph than during the falling limb. Accordingly, DOC export along the rising (Eq. (1)) and falling (Eq. (2)) limbs of the hydrograph accounted for the majority (52.9% and 47.0%) of all DOC export; only 0.13% of the DOC export occurred at baseflow.

The low proportion of DOC export at baseflow in OBS was consistent with the observation that the majority of DOC export (86%) takes place during rising and falling hydrograph in forested watersheds (Raymond and Saiers 2010). Our findings of lower DOC concentrations during the falling limb compared to the rising limb of the hydrograph may point to an initial flushing of DOC from soils, which then resulted in temporary DOC depletion (Raymond and Sayers 2010). Likewise, a clockwise DOC–discharge hysteresis has previously been attributed to a temporary depletion of the terrestrial DOC supply flushed prior to peak flow (Hornberger et al. 1994; Boyer et al. 2000; Ågren et al. 2008). Our results confirm that frequent hydrological events like storms and snowmelt determine the timing and amount of DOM fluxes (Boyer et al. 2000; Raymond and Saiers 2010) particularly in headwater streams that are well embedded in the terrestrial environment. This may be of relevance for downstream ecosystems, where the timing and composition of DOM fluxes may determine stream metabolism (Battin et al. 2008).

### Ecosystem implications

Our results suggest seasonal, diurnal, and event‐driven processes linked to hydrology, temperature, and PAR drove DOM concentration, composition, and export fluxes from an Alpine headwater stream. Seemingly, the biological processes occurring in the benthic zone imparted a dynamic autochthonous fingerprint onto an otherwise largely allochthonous DOM pool in the stream. Our findings emphasized the hyporheic zone as an interface for DOM between catchment and in‐stream processes driven to some extent by hydrology and hydrodynamic exchange between the streamwater and the hyporheic zone. The temporal dynamics of DOM in the hyporheic zone was less subject to fluctuations, which was consistent with the concept that the hyporheic zone is buffered against environmental fluctuations (Boulton et al. 1998). Furthermore, the hyporheic zone received terrestrial DOM that may be relatively resistant to microbial metabolism but which is continuously delivered. This large and steady reservoir of recalcitrant DOM in the hyporheic zone may confer stability to the stream ecosystem (*sensu* Wetzel 2001). In contrast, benthic processes sporadically delivered DOM that is more readily available for heterotrophic metabolism. Interactions between these two DOM pools as encapsulated by the notion of priming still remaining to be unveiled in stream ecosystems (Bengtsson et al. 2014).

The involvement of the various components of stream ecosystems, even including the catchment itself, in DOM fluxes is susceptible to changes in hydrology. At seasonal and diurnal scales, biological processes (e.g., primary production) and ensuing DOC concentration seem to be relevant drivers of DOM composition, whereas at the event‐driven are storms and extended baseflow controls on of DOM composition and export fluxes to downstream ecosystems. Untangling these processes and patterns is crucial to better understand how seasonal hydrology but also the hydrodynamic exchange between streamwater and the hyporheic zone likely affect DOM export fluxes as climate changes. In fact, shifts in precipitation patterns and snow pack are predicted to have major impacts on the hydrological regime of streams (Berghuijs et al. 2014) and their connectivity with the catchment and groundwater. Our study paves the way toward a better understanding of the possible consequences of such environmental changes on the DOM biogeochemistry in Alpine streams and on the downstream implications.
